# RBM47 promotes cell proliferation and immune evasion by upregulating PDIA6: a novel mechanism of pancreatic cancer progression

**DOI:** 10.1186/s12967-024-05970-6

**Published:** 2024-12-31

**Authors:** Yihui Ma, Enjie Liu, Huijie Fan, Chenfei Li, Pei Huang, Meiying Cui, Zhengyang Wang, Jing Zhou, Kuisheng Chen

**Affiliations:** 1 Department of Pathology, The First Affiliated Hospital of Zhengzhou University, No. 1, Jianshe East Road, Zhengzhou, China; 2 Department of Oncology, The First Affiliated Hospital of Zhengzhou University, Zhengzhou, China

**Keywords:** Pancreatic cancer, RBM47, PDIA6, Immune evasion

## Abstract

**Background:**

Pancreatic cancer (PC) is a lethal malignancy characterized by poor prognosis and high mortality. We found the highly expressed RNA-binding motif protein 47 (RBM47) in PC progression. The RBM47 expression was negatively correlated with natural killer (NK) cell infiltrate in PC. Moreover, RBM47 was predicted to bind to the 3′-UTR region of Protein Disulfide Isomerase Family A Member 6 (PDIA6), an oncogene of the development of PC. Therefore, we supposed that RBM47 might affect PC progression by regulating PDIA6.

**Methods:**

Bioinformatics analysis was performed to screen the candidate gene affecting PC progression using public databases. Loss- and gain-of-function effects of RBM47 on cell proliferation, tumor growth, and immune evasion were determined by CCK-8, EdU incorporation, colony formation assays, the xenogeneic tumor model, and co-culture system of PC and NK-92 cells. RBM47-RNA immunoprecipitation (RIP) followed by PCR and dual luciferase reporter assay were used to detect whether RBM47 could interact with the PDIA6 mRNA and how RBM47 would regulate the transcriptional activity of PDIA6, respectively. Simultaneous overexpression of PDIA6 in RBM47 knockdown PC cells was conducted to clarify whether PDIA6 would mediated effects of RBM47. Given the important role of cellular metabolism in cells proliferation and immune evasion, PC cells with RBM47 knockdown were subjected to metabolomics analysis to further investigate how RBM47 regulate PC progression.

**Results:**

RBM47 overexpression drove PC progression by promoting cell proliferation and xenografted tumor growth. Consistently, our results showed that RBM47 overexpression weakened sensitivity of PC cells to cytotoxic NK cells. However, RBM47 knockdown exhibited the opposite effects on proliferation and immune evasion of PC cells. RBM47 was able to bind to the 3′-UTR region of PDIA6, maintained PDIA6 mRNA stability, and increased the PDIA6 expression in PC cells. Rescue experiments supported that PDIA6 overexpression reversed the suppressing effects of RBM47 knockdown on cell proliferation and immune evasion. RBM47 knockdown significantly changed metabolites of PC cells.

**Conclusions:**

In summary, our findings demonstrate that RBM47 contributes to PC progression, which might be mediated by the upregulated PDIA6 expression and the altered cellular metabolites in PC cells, offering a potential therapeutic target for PC treatment.

**Graphical Abstract:**

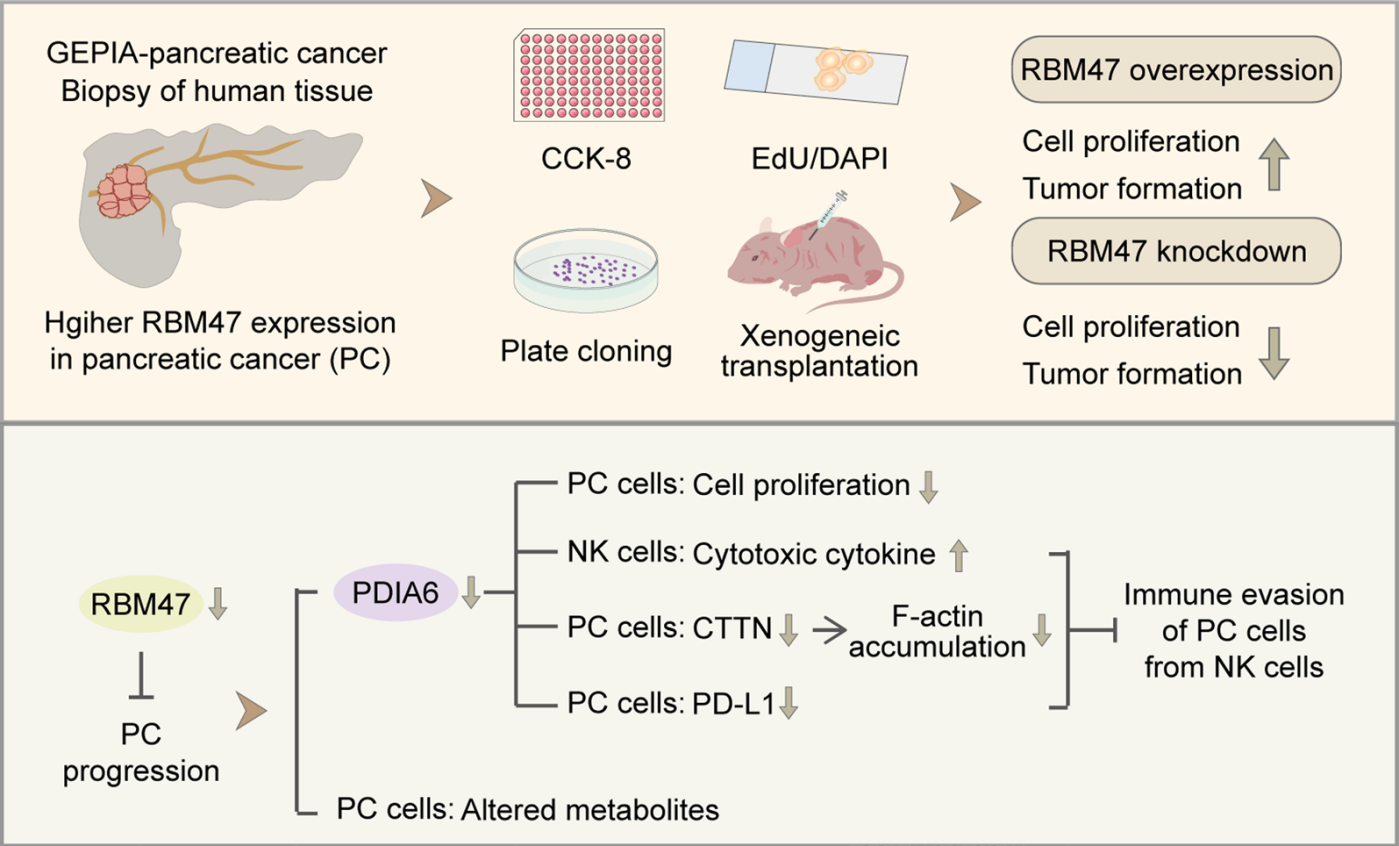

**Supplementary Information:**

The online version contains supplementary material available at 10.1186/s12967-024-05970-6.

## Introduction

Pancreatic cancer (PC) is a lethal malignancy with an overall 5-year survival rate of less than 5%, ranking the seventh cause of cancer-related deaths worldwide [1, 2]. The annual number of diagnosed PC cases has doubled worldwide over the past two decades. Aging, unhealthy lifestyle habits (such as cigarette smoking and alcohol intake), and other diseases (such as obesity and diabetes) are considered to be related to the increase in the incidence of PC [3]. The deeper position of the pancreas makes it difficult for most patients with early PC to emerge symptoms, which in turn causes most cases to be diagnosed at the later stage [4]. Surgery, radiation therapy, and chemotherapy are currently common treatment methods for PC, while surgery is only possible for the earlier PC, and therapy and chemotherapy have limited therapeutic effects due to the high resistance of PC [5]. Therefore, efforts to find a novel therapeutic target are necessary for providing new insight into PC treatment.

RNA-binding motif protein 47 (RBM47) is a RNA-binding protein (RBP), belonging to the hnRNP-R/Q-paralog family [6]. Several studies have reported the different role of RBM47 in cancer progression. Specifically, the decreased RBM47 expression was associated with aggravated migration and metastases formation of colorectal cancer cells [7]. RBM47 knockdown corresponded to the upregulated respiratory metabolism and drug resistance of lung adenocarcinoma A549 cells and promoted xenografted tumor growth in nude mice [8]. However, Soleymanjahi et al*.* suggested that intestine-specific *Rbm47*-knockout mice exhibited the weaker colitis-associated tumorigenesis compared to *Rbm47*^*fl/fl*^ counterparts after Azoxymethane-Dextran Sodium Sulfate treatment [9]. RBM47 deficiency was found to relieve proliferation, migration, and tumorigenesis of nasopharyngeal carcinoma cells whereas its overexpression showed the exact opposite trend [10]. These previous studies provide an important hint that whether RBM47 exerts cancer-promoting or cancer-suppressing effects depends on the type of cancer. There are currently no reports regarding how RBM47 affects the development of PC.

Natural killer (NK) cells are one of the components of the innate immune system and they play an important role in the immune response to tumors [11]. They could recognize and attack tumor cells by secreted effector molecules such as perforin and granzymes, thereby exerting direct cytotoxicity [12–14]. Immune synapse (IS), referring to a structured interface formed at the NK cell-tumor contact site, plays a major part in the process of NK cell-mediated tumor cell killing [15–17]. Furthermore, Gene Set Cancer Analysis (GSCA, https://guolab.wchscu.cn/GSCA/#/) database analysis revealed a negative correlation between the RBM47 expression and NK cell infiltration in PC, suggesting the possibility that RBM47 might affect PC progression by modulating immune evasion.

Protein Disulfide Isomerase Family A Member 6 (PDIA6) is a member of the protein disulfide isomerase (PDI) family that mainly exist in the endoplasmic reticulum [18]. A body of literature has uncovered that a high expression of PDIA6 was linked to development of cancers. Our previous work showed that PDIA6 promoted PC progression and its overexpression suppressed the sensitivity of PC cells to NK-92 cell cytotoxicity [19]. More importantly, GSE58380 dataset analysis indicated that RBM47 could bind to the 3′-untranslated region (UTR) of PDIA6 mRNA in breast cancer cells. Therefore, we supposed that RBM47 perhaps regulated the PDIA6 expression at the transcriptional level, contributing to PC progression by regulating PC cell escape.

In the present study, we aimed to determine effects of RBM47 on PC progression by gain- and loss-of-function experiments. Our results determined RBM47 was a participator in the development of PC. It promoted cell proliferation and immune evasion from NK cells by upregulating the PDIA6 expression. Moreover, untargeted metabolomics analysis revealed that RBM47 knockdown altered cellular metabolites of PC cells. Taken together, the current study suggests RBM47 might affect PC progression by promoting PDIA6-mediated cell proliferation and immune evasion and altering cellular metabolites of PC cells.

## Materials and methods

### Gene Expression Profiling Interactive Analysis (GEPIA) database analysis

Bioinformatics analysis for pancreatic adenocarcinoma (PAAD) derived from GEPIA database was performed to ascertain the possibly therapeutic targets affecting PC progression. The threshold for differentially expressed genes (DEGs) was |Log_2_fold change (FC)|> 1 and *p* < 0.05. Kyoto Encyclopedia of Genes and Genomes (KEGG) and Gene Ontology (GO) enrichment analysis for DEGs were performed using cluster Profiler v4.8.3 (R package). The online website (https://www.geneontology.org/) was used for the function annotation of GO terms, including Biological Process (BP) terms, Molecular Function (MF) terms, and Cellular Component (CC) terms.

### Clinical samples

Paraffin-embedded PC tissue wax blocks were collected and used for immunohistochemistry (IHC) staining. According to the IHC score, the samples were classified as low expression samples (score < 6) or high expression samples (score ≥ 6) [20]. The characteristic information of clinical samples was listed in Table S1.

### IHC staining

Fixed tissues were dehydrated and embedded in paraffin followed by 5-μm tissue section preparation. After being dewaxed and rehydrated, tissue sections were subjected to antigen retrieval and incubation of 3% hydrogen peroxide solution. Then 1% bovine serum albumin (BSA) (Cat# A602440-0050, Sangon, Shanghai, China) solution was used to block sections. Subsequently, sections were incubated with RBM47 antibody (1:100, Cat# bs-19774, Bioss, Beijing, China) at 4 °C overnight and HRP-conjugated Goat Anti-rabbit IgG (1:100, Cat# SE134, Solarbio, Beijing, China) at room temperature for 45 min. Visualization in immunohistochemistry was performed using 3,3′-diaminobenzidine (DAB) (Cat# C520017, Sangon, Shanghai, China) staining for 10 min and hematoxylin (Cat# H8070, Solarbio, Beijing, China) counterstain for 3 min. Sections were dehydrated, mounted, and imaged using a microscope (BX53, OLYMPUS, Tokyo, Japan).

### Cell culture

SW1990, AsPC-1, and NK-92 cells were procured from iCell Bioscience (Shanghai, China). SW1990 cells were cultured with Leibovitz’s L-15 medium (Cat# PM151010, Procell, Wuhan, China) supplemented with 10% fetal bovine serum (FBS) and maintained in an incubator at 37 °C with 100% air. AsPC-1 cells were cultured with Roswell Park Memorial Institute (RPMI)-1640 medium supplemented with 10% FBS and maintained in an incubator at 37 °C with 5% CO_2_. NK-92 cells were cultured with Minimum Essential Medium (MEM) α supplemented with 12.5% FBS and 12.5% horse serum and maintained in an incubator at 37 °C with 5% CO_2_.

### Construction of recombinant plasmids

The coding sequence of RBM47 was inserted into pLVX-TetOne-Puro vector (pLVX-RBM47) and the short hairpin RNAs of RBM47 (shRBM47) was inserted into Tet-pLKO-puro vector (pLKO-shRBM47). Empty pLVX-TetOne-Puro vector (pLVX-EV) and non-targeting shRNA (pLKO-shNC) were used as their respective controls. The recombinant plasmids and lentiviral packaging plasmids were co-transfected into 293 T cells to generate recombinant lentiviral particles. Subsequently, these lentiviral particles were used to infect cells to generate RBM47-overexpressing or -knockdown SW1990 and AsPC-1 cells. After infection, 2 μg/ml puromycin was added into culture medium to obtain stably infected cells, which were stimulated by 1 μg/ml of Doxycycline (Dox) for 24 or 48 h to induce RBM47 overexpression and knockdown in cells. For PDIA6 overexpression in RBM47-knockdown AsPC-1 cells, a vector containing the coding sequence of PDIA6 (OE-PDIA6) was constructed and cell transfection was achieved using Lipofectamine 3000. Then, cell were treated with Dox (1 μg/ml) and cultured in an incubator at 37 °C with 5% CO_2_ for 48 h. For CTTN overexpression in PC cells, a vector containing the coding sequence of CTTN (OE-CTTN) was constructed and used to transfect cells.

### Cell proliferation

Cell proliferation was determined by cell counting kit (CCK)-8, EdU incorporation, and colony formation assay. For CCK-8 assay, cells were seeded into 96-well plates (5 × 10^3^ cells/per well) and cultured in an incubator at 37 °C with 5% CO_2_ for 0, 24, 48, or 72 h. NK-92 cells and PC cells were mixed in the ratio of 2:1, 1:1, or 1:2, and seeded into a 96-well plates (5 × 10^3^ cells/per well), followed by the treatment of 1 μg/ml Dox and 48 h of cultivation. Then, 10 μl of CCK-8 (KGA317, KeyGEN, Nanjing, China) solution was added to each well to incubate cells for 2 h. The optical density (OD) value was measured by a microplate reader (800TS, BioTek, Winooski, USA) at 450 nm.

EdU incorporation assay was performed by flow cytometry using E-Click EdU Cell Proliferation Flow Cytometry Assay Kit (Cat# E-CK-A370, Elabscience, Wuhan, China). Briefly, cells were collected and resuspended with phosphate buffer saline (PBS) containing 1% BSA. The cell suspension was centrifuged, and the supernatant was discarded. Cells were resuspended with PBS containing 4% paraformaldehyde and incubated at room temperature in the dark for 15 min. Cells were then resuspended with PBS containing 1% BSA and centrifuged to remove the supernatant, which was repeated twice. Next, cells were incubated with PBS containing 1% Saponin at room temperature for 20 min. Cells were collected and treated with Click Reaction Buffer at room temperature in the dark for 30 min. Subsequently, cells were treated with PBS containing 1% Saponin. After being centrifuged, cells were mixed with PBS containing 1% BSA and subjected to flow cytometry analysis (NovoCyte, Agilent, Santa Clara, USA).

For colony formation assay, cells were seeded into a culture dish (300 cells/per dish) and 1 μg/ml Dox was added simultaneously. Then, culture dishes were placed in the incubator. After 2 weeks of cultivation, colonies were fixed with 4% paraformaldehyde and stained with Wright-Giemsa staining (Cat# KGA227, KeyGEN, Nanjing, China) for 5 min. The number of colonies was counted under a microscope (IX53, OLYMPUS, Tokyo, Japan). Cell clusters with 50 or more cells are recorded as one colony. Colony formation rate was calculated by the following formula: number of colonies/inoculated cells number × 100%.

### RNA isolation and quantitative real time-PCR (qRT-PCR)

RNA was extracted from cells using TRIZOL (Cat# RP1001, BioTeke, Beijing, China) and chloroform, followed by precipitation using isopropyl alcohol. After being washed with ethanol solution, RNA was dissolved in RNase-free water and its concentration was quantified by spectrophotometer (NANO2000, Thermo Fisher, Waltham, USA). Then, reverse transcription was performed by Oligo(dT)15 and BeyoRT II M-MLV reverse transcriptase (Cat# D7160L, Beyotime, Shanghai, China) to obtain cDNA, which was subjected to qRT-PCR along with primers and SYBR GREEN on fluorescence quantifier (Exicycler 96, BIONEER, Daejeon, Korea). Primer sequences are as follows: RBM47 F, 5′-AGCCGCTGTCATTCCCACT-3′; RBM47 R, 5′-GGTATGTAGCCTGCGTATCCT-3′; PDIA6 F, 5′-GTCCCAGCCAACATCAGC-3′; PDIA6 R, 5′-CCAGACCGGGTGCATTAG -3′; CTTN F, 5′-ATTCGGTGTTCAGTCGG-3′; CTTN R, 5′-ACCTGGGTGACATCCTC-3′; GAPDH F, 5′-GACCTGACCTGCCGTCTAG-3′; GAPDH R, 5′-AGGAGTGGGTGTCGCTGT-3′.

### Lactatedehydrogenase (LDH) release assay

The cytotoxic activity of NK cells against PC cells was determined by the LDH release in the supernatant of the culture medium using LDH cytotoxicity detection Kit (Cat# CT0027, LEAGENE, Huaibei, China) as previously described [21]. Briefly, NK cells were co-cultured with PC cells with the E:T ratio of 2:1, 1:1, and 1:2, which were treated with Dox for 48 h. Then the supernatant was removed in a new 96-well plate, and LDH Assay Buffer and NAD Buffer were added to incubate samples at 37 °C for 15 min. Phenylhydrazine solution was used to treat samples at 37 °C for another 15 min. Then alkaline chromogenic solution and distilled water were added and mixed with the reaction system, which was placed for 5 min. The absorbance of samples was detected using a microplate reader (Synergy H1, BioTek, Winooski, USA). The percentage of cytotoxicity was calculated according to the formula: (experimental − target spontaneous)/(maximum—spontaneous) × 100%.

### Phalloidin staining

Cells were plated on coverslips for histological staining. After being fixed with 4% paraformaldehyde, cells were permeated with 0.1% tritonX-100 (Cat# ST795, Beyotime, Shanghai, China) and treated with TRITC Phalloidin (Cat# CA1610, Solarbio, Beijing, China) to stain cellular F-actin. DAPI was used to stain nuclei. Cells were mounted by anti-fluorescence quencher medium (Cat# S2100, Solarbio, Beijing, China) and viewed using a microscope (BX53, OLYMPUS, Tokyo, Japan).

### Immunofluorescence staining

Tumor tissues resected from nude mice were fixed, dehydrated, and embedded in paraffin. Subsequently, 5-μm tissue sections were prepared for immunofluorescence staining. Specifically, following dewaxing and rehydration, sections were exposed to citrate buffer for heat-induced antigen retrieval and 1% BSA solution for blocking. Subsequently, primary antibodies (1:100) were used to treat sections at 4 °C overnight, followed by incubation of Cy3-conjugated goat anti-rabbit IgG (1:200, Cat# SA00009-2, Proteintech, Wuhan, China) at room temperature for 60 min. After being stained with DAPI and mounted by an anti-fluorescence quencher medium (Cat# S2100, Solarbio, Beijing, China), sections were photographed by a microscope (BX53, OLYMPUS, Tokyo, Japan). Commercially sourced of primary antibodies are as follows: RBM47 (Cat# bs-19774, Bioss, Beijing, China) and Ki67 (Cat# A20018, ABclonal, Wuhan, China).

NK cells were labelled with CellTracker Red CMTPX dye (Cat# MX4109, Maokangbio, Shanghai, China) and co-cultured with PC cells with the E:T ratio of 2:1 [22]. After being incubated with Dox for 48 h, cells were subjected to immunofluorescence staining. In brief, fixed cells were permeated with 0.1% tritonX-100 and blocked with 1% BSA. Subsequently, cells were incubated with F-actin antibody (1:100, Cat# ab205, Abcam, Shanghai, China) at 4 °C overnight, and its secondary antibody goat anti-mouse IgG (1:200, Cat# SA00003-1, Proteintech, Wuhan, China) at room temperature for 1 h. After being stained with DAPI, cells were treated with anti-fluorescence quencher medium (Cat# S2100, Solarbio, Beijing, China) and imaged using a confocal microscope (NiKon, Tokyo, Japan).

### Western blot

Total protein samples were extracted from cells using the mixture of RIPA buffer (Cat# R0010, Solarbio, Beijing, China) and PMSF (Cat# P0100, Solarbio, Beijing, China). After being quantified by BCA Protein Assay Kit (Cat# PC0020, Solarbio, Beijing, China), equal amounts of protein samples were loaded on and separated by 10% SDS–polyacrylamide gels and transferred to PVDF membranes (Cat# IPVH00010, Millipore, Temecula, USA). Next, blocked PVDF membranes were incubated with primary antibodies at 4 °C overnight, followed by visualization of secondary antibodies at 37 °C for 1 h and ECL luminescence (Cat# PE0010, Solarbio, Beijing, China) for 5 min. Commercial sources of antibodies are as follows: RBM47 antibody (1:1000, Cat# bs-19774, Bioss, Beijing, China), CTTN antibody (1:3000, Cat# 11381-1-AP, Proteintech, Wuhan, China), PDIA6 antibody (1:500, Cat# A7055, ABclonal, Wuhan, China), PD-L1 antibody (1:1000, Cat# 17952-1-AP, Proteintech, Wuhan, China), goat anti-rabbit IgG (1:3000, Cat# SE134, Solarbio, Beijing, China). GAPDH antibody (1:10,000, Cat# 60004-1-Ig, Proteintech, Wuhan, China) was used for normalization and its corresponding secondary antibody was goat anti-mouse IgG (1:3000, Cat# SE131, Solarbio, Beijing, China).

### Co-immunoprecipitation (IP) assay

The whole protein was extracted from PC cells using Native lysis Buffer (Cat# R0030, Solarbio, Beijing, China) and quantified. AminoLink coupling resin was added to the Pierce spin column, which was incubated with CTTN antibody or the negative control IgG antibody for 120 min. Then, the whole protein sample was added to the column, which was placed at room temperature and gently shaken for 2 h. The column was treated with elution buffer solution for 5 min and the flow-through was collected for immunoblotting. Protein samples were separated by SDS-PAGE electrophoresis and transferred to PVDF membranes. After being blocked, membranes were incubated with CTTN antibody (1:3000, Cat# 11381-1-AP, Proteintech, Wuhan, China) or F-actin antibody (1:1000, Cat# ab205, Abcam, Shanghai, China) at 4 °C overnight, and the corresponding secondary antibody goat-anti rabbit IgG (1:3000, Cat# SE134, Solarbio, Beijing, China) or goat-anti mouse IgG (1:3000, Cat# SE131, Solarbio, Beijing, China), followed by ECL luminescence.

### Enzyme linked immunosorbent assay (ELISA)

The cell supernatant was collected and used for ELISA. Human IFN-γ ELISA Kit (Cat# EK180), Human Granzyme B ELISA Kit (Cat# EK158), and Human MIP-1α ELISA Kit (Cat# EK161) were purchased form Liankebio (Hangzhou, China). The specific procedures were conducted following the manufacturer’s protocols.

### PD1 and PD-L1 interaction assay

PC cells were incubated with Dox for 48 h, followed by the treatment of FITC (Cat# I6141, Macklin, Shanghai, China)-labelled PD-1 (Cat# HY-P73344, Med Chem Express, Shanghai, China) [23]. After 12 h, the binding between PD-1 and PD-L1 at PC cells were visualized using a microscope.

### Xenograft tumor model

Female BALB/c nude mice (6 weeks) were purchased from Huachuang Sino (Taizhou, China) and housed at 22 ± 1 °C in 12:12 light/dark cycles for 1 week of adaptive feeding. Later, cells in the logarithmic growth phase were collected and subcutaneously injected into mice (1 × 10^7^ cells/per mouse). After the formation of visible tumors, mice were fed with drinking water containing Dox (1 mg/ml). The tumor volume was measured every 3 days, which lasted for 30 days. Nude mice were euthanized and then tumor bodies were stripped, weighed, and fixed for subsequent experiments.

### RNA-binding protein immunoprecipitation (RIP)

EZ-Magna RIP™ RNA-Binding Protein Immunoprecipitation Kit (Cat# 17-701) was purchased from Millipore (Billerica, Massachusetts, USA) for RIP assay. Cell lysate was prepared using RIP lysis buffer and it was centrifuged to collect the supernatant. Magnetic beads were suspended with RIP wash buffer and incubated with antibody to obtain the magnetic bead-antibody complex. The supernatant of cell lysate was incubated with RIP immunoprecipitation buffer containing the magnetic bead-antibody complex at 4 °C overnight. The non-incubated supernatant of cell lysate was used the “Input”. The immunoprecipitate was washed with RIP wash buffer and resuspended in proteinase K buffer to remove protein. RIP wash buffer, 10% SDS, and proteinase K buffer were used to treat Input samples. The immunoprecipitated-RNA was purified and transcribed into cDNA, which was subjected to PCR analysis.

### Luciferase reporter assay

The 293 T cells were purchased from iCell Bioscience (Shanghai, China) and cultured with Dulbecco’s modified eagle medium (Cat# G4510, Servicebio, Wuhan, China) supplemented with 10% FBS in an incubator at 37 °C with 5% CO_2_. The 3′-UTR region of PDIA6 mRNA was inserted into the pGL3-Basic vector, which was transfected into 293 T cells along with RBM47-flag overexpression vector or empty vector. After 48 h of transfection, cells were lysed and the luciferase activity was detected using Dual Luciferase Reporter Assay Kit (Cat# KGAF040, KeyGEN, Nanjing, China).

### Untargeted metabolomics analysis

After being treated with Dox for 48 h, infected PC cells were washed with cold phosphate buffer saline solution for three times and collected by centrifugation. Then cells were subjected to untargeted metabolomics analysis. The threshold for differentially differential metabolites was Variable Importance in the Projection (VIP) > 1, p < 0.05, and FC ≥ 2 or FC ≤ 0.5.

### Statistical analysis

Data were expressed as the mean ± standard deviation (SD) and statistically analyzed using the Graphpad Prism 9 software. The Unpaired-t or Mann–Whitney test was used for comparisons between two groups for cells, while Chi-square test for clinical samples. The one-way ANOVA with Tukey test were used for comparisons among three or more groups. The differences was considered significant when *p* < 0.05.

## Results

### RBM47 might be a participant in PC progression

**Fig. 1:**
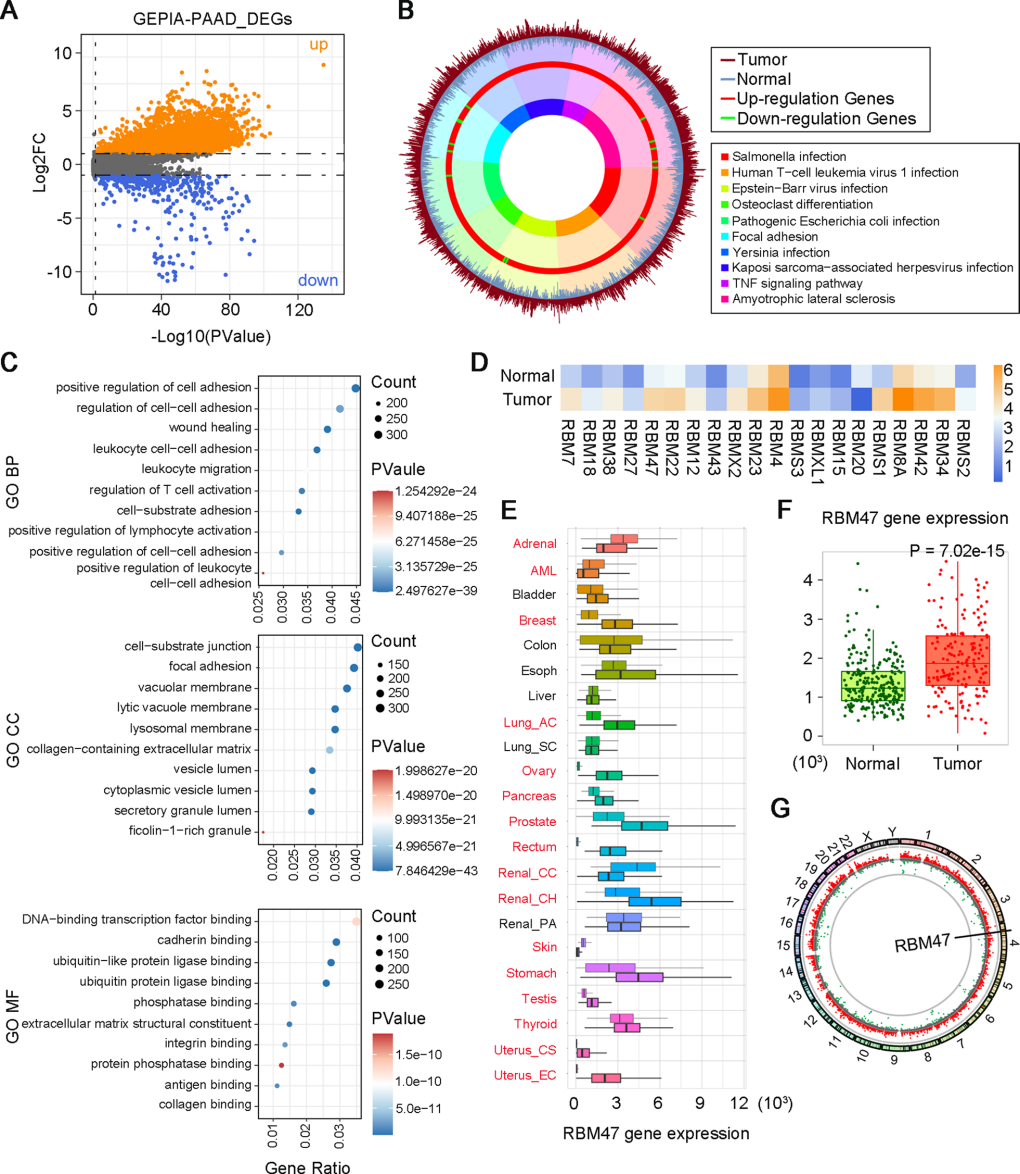
High RNA Binding Motif Protein 47 (RBM47) expression might be associated with pancreatic cancer (PC) progression. **A** The volcano plot of differentially expressed genes (DEGs) analyzed from pancreatic adenocarcinoma (PAAD) dataset in Gene Expression Profiling Interactive Analysis (GEPIA) database. **B** Enriched Kyoto Encyclopedia of Genes and Genomes (KEGG) pathways of DEGs analyzed from PAAD dataset in GEPIA database. **C** Enriched Gene Ontology (GO) annotations of DEGs analyzed from PAAD dataset in GEPIA database. **D** The heat map of DEGs from RBM family analyzed from PAAD dataset in GEPIA database. **E** Pan-cancer analysis of RBM47 expression in multiple cancer types analyzed from TNMplot. **F** The RBM47 expression in PAAD analyzed from TNMplot. **G** The circus plot of the location of RBM47 on chromosomes

To find a new therapeutic target for PC treatment, we analyzed the dataset of PAAD, the most common pathologic type of PC [24], from the GEPIA database to identify DEGs. The volcano plot showed significantly up-regulated (orange scatter points) and down-regulated genes (blue scatter points) in PAAD samples compared with normal samples (Fig. 1A). Then, KEGG and GO enrichment analysis were performed to explore potential biological functions of DEGs. In the plot of KEGG enrichment analysis, the up-regulated and down-regulated DEGs were marked using the red and green color respectively, in the third layer. A heavier proportion of up-regulated DEG could be observed, which demonstrated that top 10 of KEGG pathways were mainly enriched by up-regulated DEGs (Fig. 1B). Based on that, we narrowed down the screening range of the new therapeutic target to upregulated DEGs. GO analysis of DEGs, including top 10 of biological process (BP), cell component (CC), and molecular function (MF), was presented in Fig. 1C. In the MF category, DNA-binding transcription factor binding was one of the major GO terms. RBM family proteins have been reported to transcriptionally regulate the expression of DNA-binding transcription factors and affect their translation. Notably, RBM family proteins involve in a series of RNA processes, such as splicing, translation, and RNA silencing [25]. Dysregulated RNA translation is usually occurred in cancers [26]. Considering the important function of RBM proteins in RNA processing, we further identified the list of DEGs in the RBM family. Expression profiles of 20 RBM family DEGs were visualized in the heat map (Fig. 1D). NK cells are crucial for anti-tumor immunity [27]. Therefore, we further analyzed the correlation between these DEGs and NK cell infiltration in PAAD using the GSCA website (https://guolab.wchscu.cn/GSCA/#/). The results showed that only RBM38, RBM47, RBM23, RBMS3, RBM20, and RBM8A were significantly correlated with NK cell infiltration in PAAD. By searching the literature, we found that the RBM38’s role in the development of PC has been reported, and RBM47 and RBMS3 were closely associated with tumor immunity. RBM47 had a higher Log_2_FC value in the GEPIA-PAAD dataset compared with RBMS3. Based on that, RBM47 was selected for further analysis. Next, we performed a pan-cancer analysis of RBM47 expression by the TNMplot web tool. The results showed that the expression profile of RBM47 varied in different types of cancers. Of note, it has a significantly higher expression in pancreatic tumors than normal samples (Fig. 1E, F). The circus plot indicated that RBM47 is located on the chromosome 4 (Fig. 1G). The above results suggested that the upregulated RBM47 expression might be closely related to the development of PC. Therefore, we collected tumor tissues from patients with PC and performed IHC staining. Representative images and the IHC score of PC tissues with low or high RBM47 expression were displayed in Fig. S1A and B, respectively. Subsequently, the clinicopathological characteristics of patients with high or low RBM47 expression were analyzed. The results showed that the RBM47 expression was significantly associated with the T stage of PC (Table S1). Taken together, we supposed that RBM47 might be a candidate gene affecting the development of PC.

### RBM47 promoted cell proliferation of PC cells

**Fig. 2:**
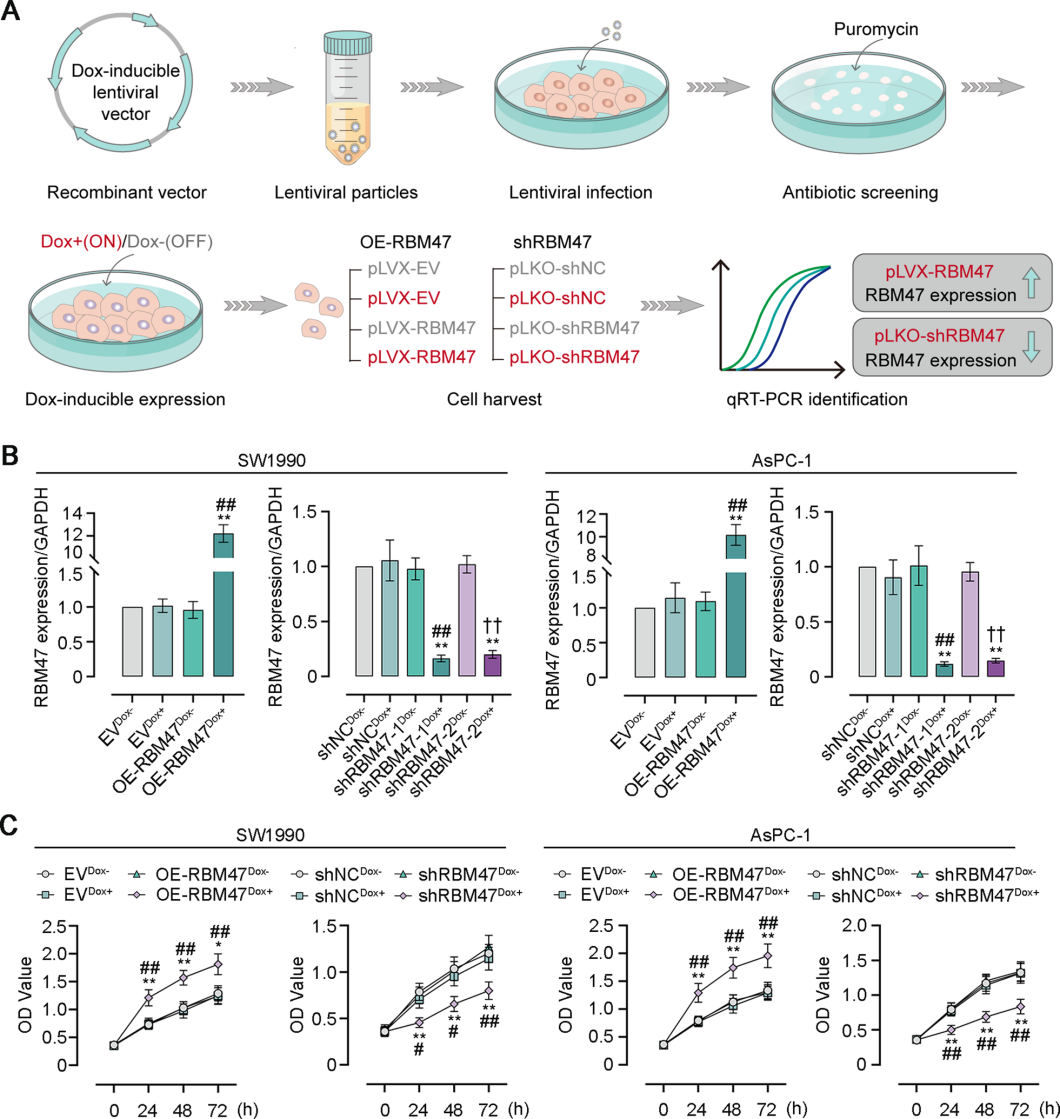
RBM47 increased cell viability of PC cells. **A** The schematic diagram of Doxycycline (Dox)-inducible lentivirus vectors. **B** The mRNA level of RBM47 expression in PC cells (normalized by GAPDH). **C** The CCK-8 assay for cell viability of PC cells. *p < 0.05, **p < 0.01 versus EV^Dox−^ or shNC^Dox−^ groups. ^#^p < 0.05, ^##^p < 0.01 versus OE-RBM47^Dox−^ or shRBM47-1^Dox−^, ^†^p < 0.05, ^††^p < 0.01 versus shRBM47-2^Dox−^

**Fig. 3:**
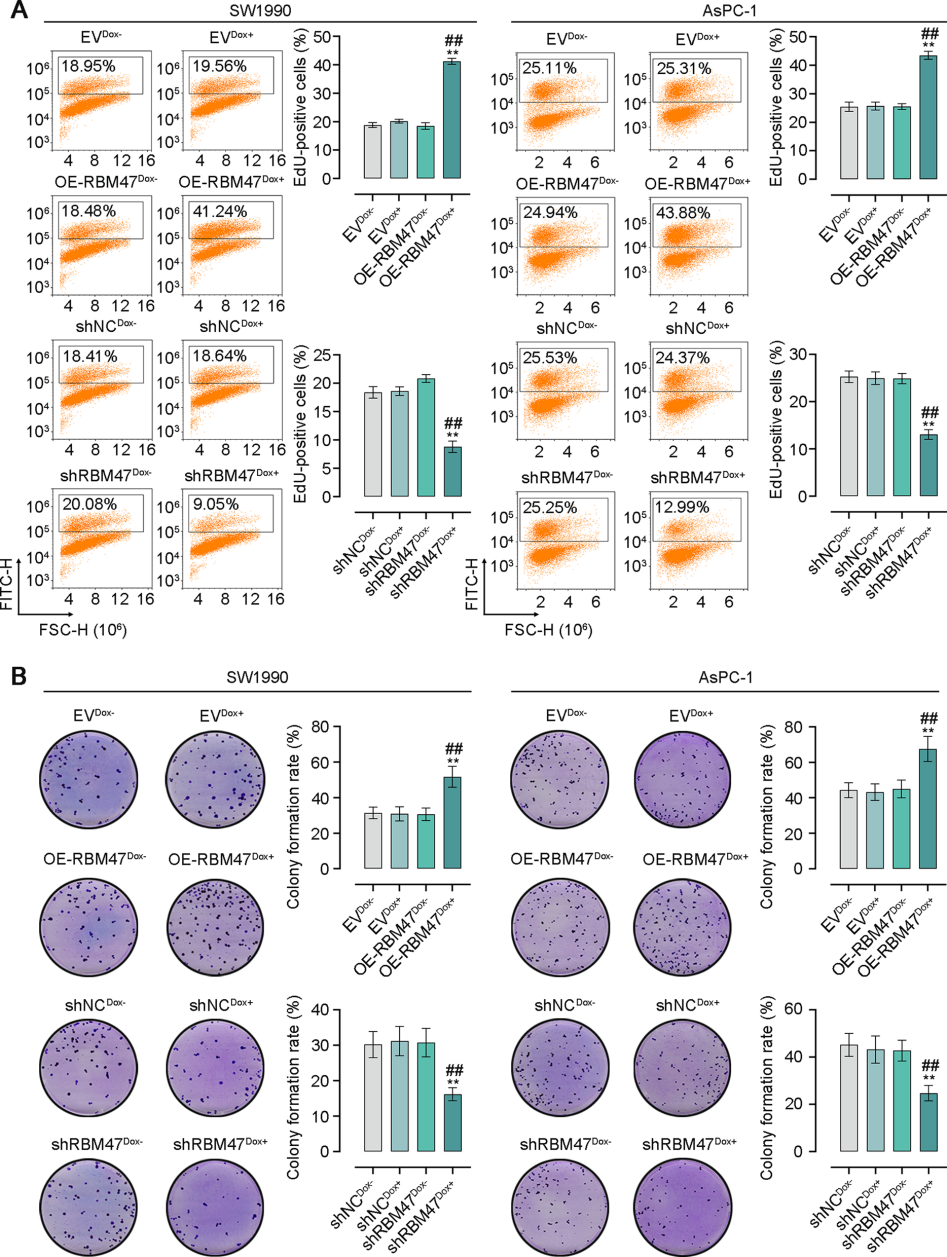
RBM47 enhanced cell proliferation of PC cells. **A** Flow cytometry analysis for the percentage of EdU-positive PC cells. **B** The plate cloning experiment for cell proliferation of PC cells. *p < 0.05, **p < 0.01 versus EV^Dox−^ or shNC^Dox−^ groups. ^#^p < 0.05, ^##^p < 0.01 versus OE-RBM47^Dox−^ or shRBM47^Dox−^

To investigate the function of RBM47 expression in PC progression, we employed lentiviral vectors expressing Dox-inducible RBM47 or shRBM47 to infect PC SW1990 and AsPC-1 cells for RBM47 overexpression or knockdown. After infection, puromycin was added to culture medium for screening stably infected cells. Subsequently, the overexpression or knockdown of RBM47 in PC cells was initiated by Dox treatment (Fig. 2A). The RBM47 mRNA level detected by qRT-PCR confirmed the efficiency of overexpression or knockdown in PC cells as demonstrated by the increased RBM47 expression in pLVX-RBM47-infected PC cells with Dox treatment. However, the RBM47 expression in pLKO-RBM47-infected PC cells with Dox treatment pLVX-RBM47-infected displayed the completely opposite trend (Fig. 2B). CCK-8 assay was then conducted after 24, 48, or 72 h of lentiviral infection to indicate cell proliferation. We found that RBM47 overexpression effectively promoted cell viability of PC cells while it knockdown inhibited cell viability of PC cells (Fig. 2C). Next, EdU incorporation of PC cells was assessed by flow cytometry analysis to quantify the percentage of EdU-positive cells. The results showed that the percentage of EdU-positive PC cells was elevated after RBM47 overexpression but decreased after RBM47 knockdown (Fig. 3A). Colony formation rate obtained from plate cloning experiment also supported that RBM47 overexpression promoted cell proliferation (Fig. 3B).

### RBM47 encouraged tumor growth in nude mice

**Fig. 4:**
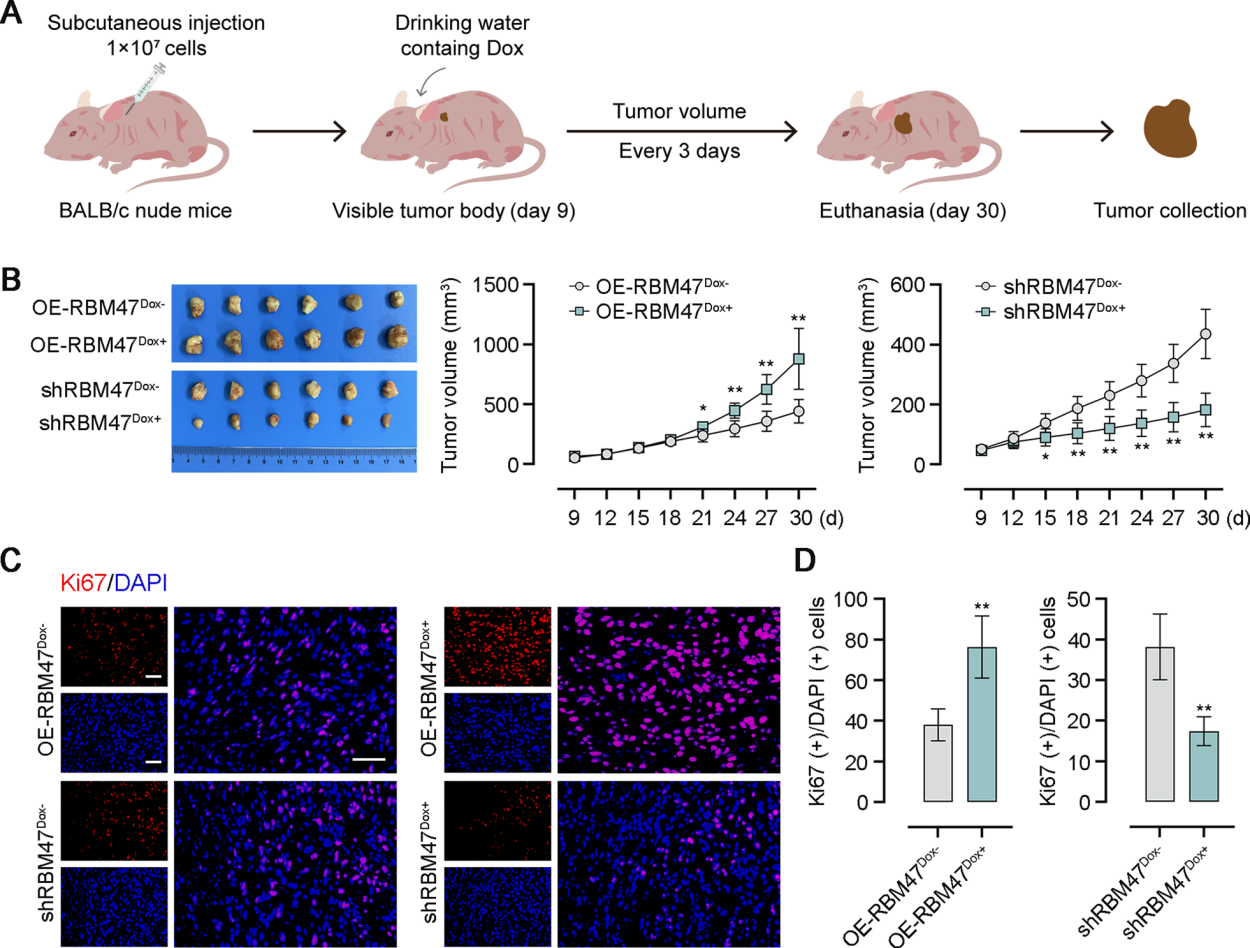
RBM47 contributed to tumor formation in xnegraft transplantation experiments. **A** The schematic diagram of xenografted tumor growth. **B** The photos of tumors dissected from nude mice and the tumor volume. **C** Immunofluorescence staining for Ki-67 of tumor tissues. Scale bar = 50 μm. **D** Quantification of the percentage of Ki67(+)/DAPI(+) cells in **C**. *p < 0.05, **p < 0.01 versus OE-RBM47^Dox−^ or shRBM47^Dox−^

Xenograft tumor models were constructed by subcutaneous injection of RBM47-overexpressing or -knockdown PC cells into nude mice to verify effects of RBM47 on tumor formation. We observed tumor formation on the 9th day after subcutaneous injection. Starting from this day, Dox was added to the drinking water of mice and the tumor volume was measured every 3 days until the 30th day (Fig. 4A). The measurement results showed that mice injected with RBM47-overexpressing PC cells formed the bigger tumor body from the 21th day. Conversely, xenotransplantation of RBM47-knockdown PC cells led to smaller tumor bodies in mice from the 15th day (Fig. 4B). The proliferation marker protein Ki67 in tumor tissues was immunofluorescence staining for assessing cell proliferation. Representative images of immunofluorescence staining and quantitative analysis showed that RBM47 overexpression increased the percentage of Ki67-postive cells and its knockdown exhibited the opposite effect in tumor tissues of nude mice (Fig. 4C, D).

### RBM47 facilitates the evasion of PC cells from NK cell-mediated tumor killing

**Fig. 5:**
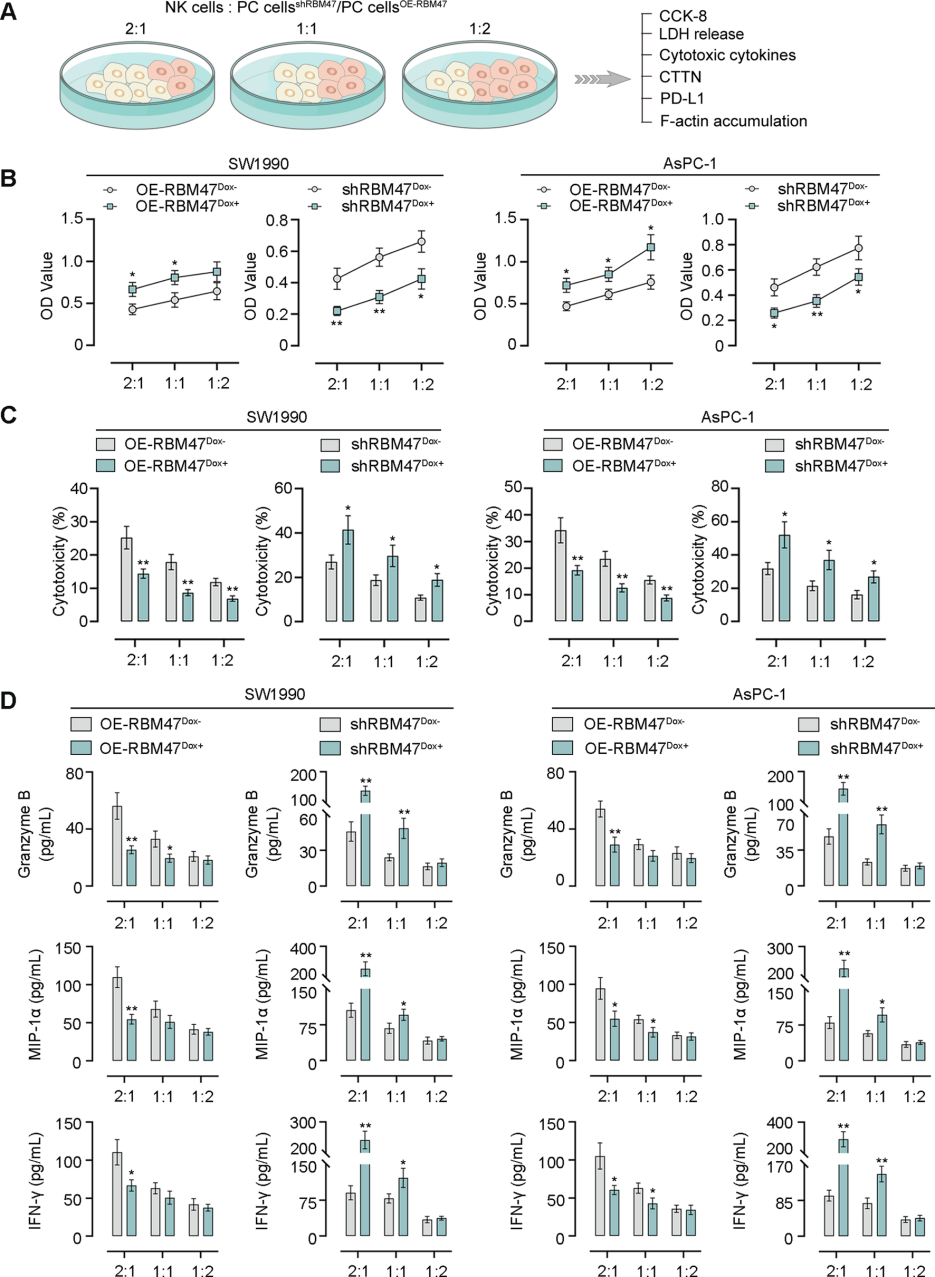
RBM47 overexpression protected PC cells from NK cells-mediated tumor cell killing. **A** The schematic diagram of co-culture of NK cells and PC cells. **B** The CCK-8 assay for cell viability of PC cells. **C** The LDH release assay for cytotoxic activity of NK cells. **D** The content of Granzyme B, MIP-1α, IFN-γ in supernatant of co-culture system NK and PC cells. *p < 0.05, **p < 0.01 versus OE-RBM47^Dox−^ or shRBM47^Dox−^

**Fig. 6:**
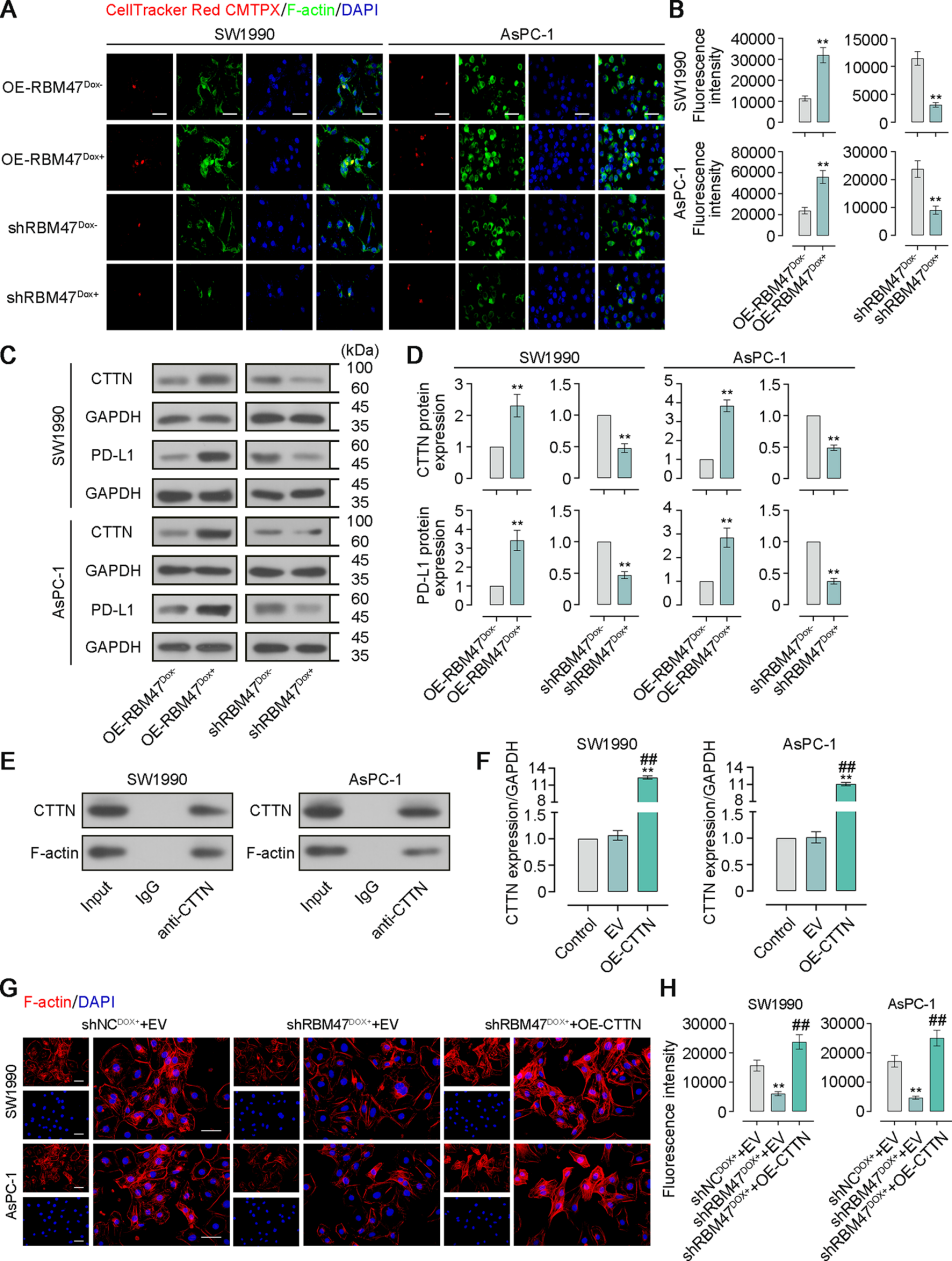
RBM47 overexpression promoted F-actin aggregation and the binding between PD-1 and PL-1. **A** CellTracker Red CMTPX-labelled NK cells and immunofluorescence staining for F-actin of PC cells. Scale bar = 50 μm. **B** Quantification of fluorescence intensity in **A**. **C** Western blot for CTTN and PD-L1 expression. **D** Quantification of the protein levels of CTTN and PD-L1 expression in **C**. **E** Co-IP assay for determine the interaction between CTTN and F-actin. **F** The mRNA level of CTTN expression in PC cells (normalized by GAPDH). **G** Rhodamine-labeled phalloidin staining for F-actin of PC cells. Scale bar = 50 μm. **H** Quantification of fluorescence intensity in **G**. **p < 0.01 versus OE-RBM47^Dox-^, shRBM47^Dox-^, or Control; ^##^p < 0.01 versus EV

Through GSCA database analysis, we found that the RBM47 expression had a negative correlation between NK cell infiltrate in PAAD (Fig. S2). Thus, we sought to further elucidate that how RBM47 affects immune evasion of PC cells. NK-92 cells were co-cultured with PC cells with the effector (E):target (T) ratio of 2:1, 1:1, or 1:2, followed by CCK-8 assay, NK cell cytotoxicity assay, cytotoxic cytokine detection, the CTTN and PD-L1 expression detection, and the visualization of F-actin accumulation (Fig. 5A). The results of CCK-8 assay showed that RBM47 overexpression promoted the resistance of PC cells to NK cells while its knockdown made PC cells more susceptible to NK cell cytotoxicity (Fig. 5B). Cytotoxic activity of NK cells was detected by the LDH release in the supernatant of the culture medium. RBM47 overexpression inhibited NK cell cytotoxicity against PC cells, but its knockdown enhanced NK cell cytotoxicity (Fig. 5C). Activated NK cells secreted key effectors such as Granzyme B, MIP-1α, and IFN-γ to enhance tumor killing function. We observed that, when the E:T ratio is 2:1, RBM47 overexpression suppressed concentrations of Granzyme B, MIP-1α, and IFN-γ in the supernatant of co-culture system while RBM47 knockdown showed an opposite trend (Fig. 5D). F-actin accumulation at immune synapses formed during the contact between NK cells and tumor cells was found to involve the immune evasion of tumor cells. Subsequently, PC cells were co-cultured with CellTracker Red CMTPX-labelled NK cells with the E:T ratio of 2:1 and stained by immunofluorescence for F-actin. Confocal microscopy images and quantitative analysis of fluorescence intensity showed that RBM47 overexpression promoted F-actin accumulation at the contact between NK cells and tumor cells. However, RBM47 knockdown exhibited the opposite trend (Fig. 6A, B). CTTN could interact with F-actin and promote its stability and aggregation [28, 29]. In addition, PD-L1 binds to PD-1 and thereby contributes to the immune escape of cancer cells [30]. By performing western blot assay and quantitative analysis, we found that RBM47 overexpression increased protein expression of CTTN and PD-L1 in PC cells. However, RBM47 knockdown decreased their expression (Fig. 6C, D). Then, we performed the Co-IP assay, and the results showed that CTTN could interact with F-actin in PC cells (Fig. 6E). CTTN overexpression in PC cells was achieved by cell transfection of the corresponding overexpression plasmid. The significantly increased CTTN mRNA expression exhibited the effective CTTN overexpression in PC cells (Fig. 6F). Images of phalloidin staining and quantitative analysis of fluorescence intensity showed that CTTN overexpression reversed the suppressing-effect of RBM47 knockdown on F-actin aggregation (Fig. 6G, H). Next, FITC-labelled PD-1 was used to treat PC cells to determine whether RBM47 would regulate the binding between PD-1 and PD-L1. Representative images and quantitative analysis of fluorescence intensity showed that RBM47 overexpression promoted the binding between PD-1 and PD-L1 in PC cells, but RBM47 knockdown had the opposite effect (Fig. S3A, B).

### RBM47 sustains the mRNA stability of PDIA6 by binding to its 3′-UTR region

**Fig. 7:**
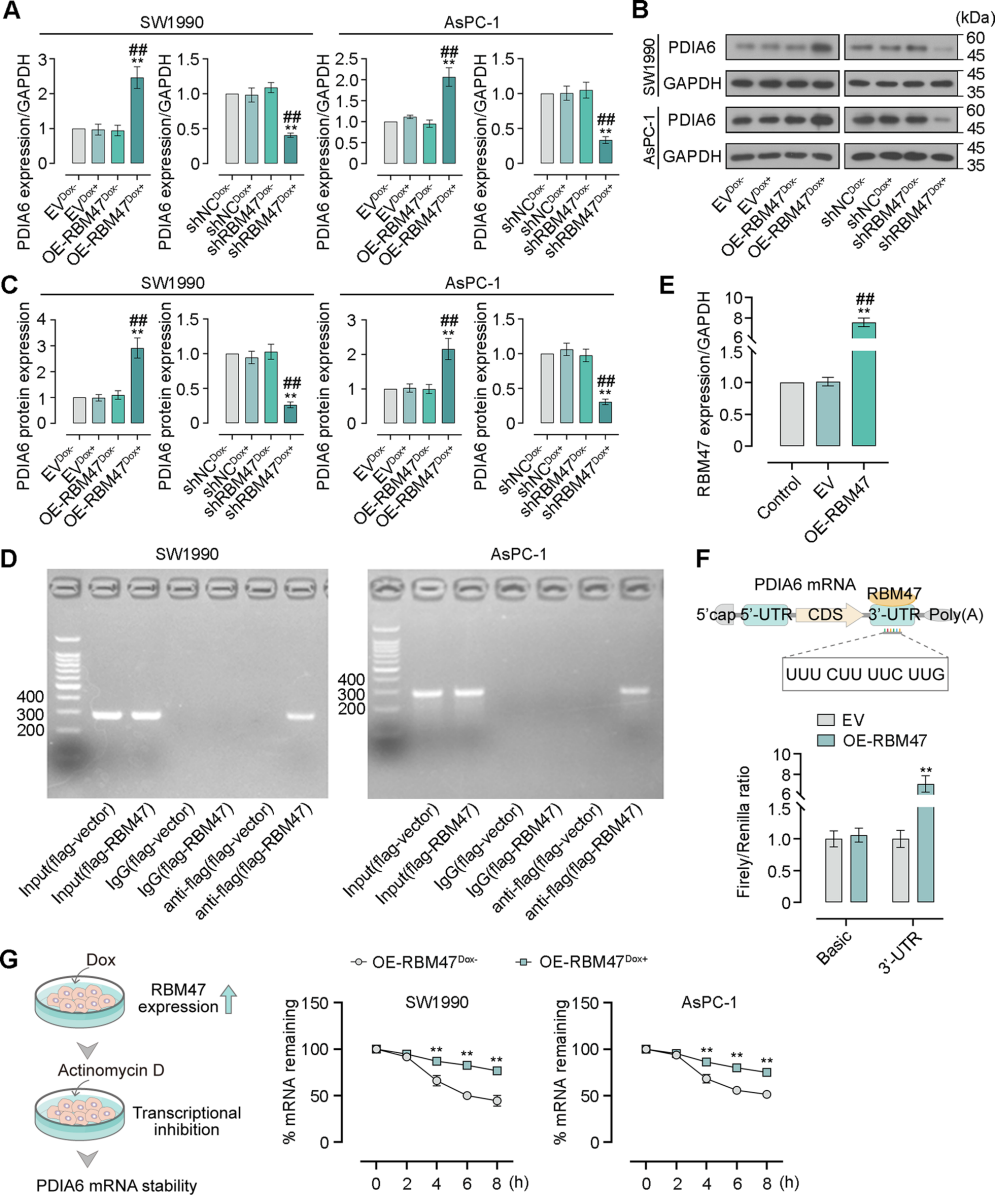
RBM47 could regulate PDIA6 mRNA stability by binding to its 3′-UTR region. **A** The mRNA level of PDIA6 expression in PC cells (normalized by GAPDH). **B** Western blot for PDIA6 expression. **C** Quantification of the protein level of PDIA6 expression in B. **D** RNA-binding protein immunoprecipitation (RIP) assay for verifying the interaction between RBM47 and the PDIA6 mRNA. **E** The mRNA level of RBM47 expression in 293 T cells (normalized by GAPDH). **F** The schematic diagram of the binding between RBM47 and the PDIA6 mRNA. Dual luciferase reporter assay for determining the effect of RBM47 overexpression on relative luciferase activity (firefly/renilla) of the 3′-UTR sequences of the PDIA6 mRNA. **G** The schematic diagram and percentage of remaining PDIA6 mRNA in PC cells after Actinomycin D treatment (normalized by GAPDH). For Fig. 7A-C, *p < 0.05, **p < 0.01 versus EV^Dox−^ or Control; ^##^p < 0.01 versus EV, OE-RBM47^Dox−^ or shRBM47^Dox−^ For **F, G**, **p < 0.01 versus EV or OE-RBM47^Dox−^

Our previous work has investigated the function of PDIA6 in PC progression. Notably, RBM47 was predicted to bind to the 3′-UTR region of PDIA6. For this reason, we therefore tested this prediction in PC cells. A validation of gene expression by qRT-PCR and western blot indicated that RBM47 overexpression markedly increased the PDIA6 expression at both mRNA and protein levels. On the contrary, its knockdown markedly decreased the PDIA6 expression level (Fig. 7A–C). The RIP assay supported the existence of PDIA6 in RBM47-RNA binding complex (Fig. 7D). To further understand the regulatory relationship between RBM47 and PDIA6, 293 T cells were co-transfected with RBM47-overexpressing vector or empty vector together with a luciferase reporter plasmid (pGL3-Basic or pGL3-PDIA6-3′-UTR). The increased RBM47 mRNA level in the OE-RBM47 group compared with the EV and control group verified the effective transfection of RBM47-overexpressing vector or empty vector in 293 T cells (Fig. 7E). RBM47 overexpression does not affect the luciferase activity of pGL3-Basic. However, the luciferase activity of pGL3-PDIA6-3′-UTR was significantly raised after RBM47 overexpression (Fig. 7F). The 3′-UTR region have a great influence on the maintenance of RNA stability. Hence, the transcription of lentiviral vector-infected PC cells with Dox induction were blocked using Actinomycin D treatment for 0, 2, 4, 6, or 8 h. The results showed that RBM47 overexpression was conducive to sustaining the PDIA6 mRNA stability (Fig. 7G).

### PDIA6 mediates effects of RBM47 on cell proliferation and immune evasion of PC cells

**Fig. 8:**
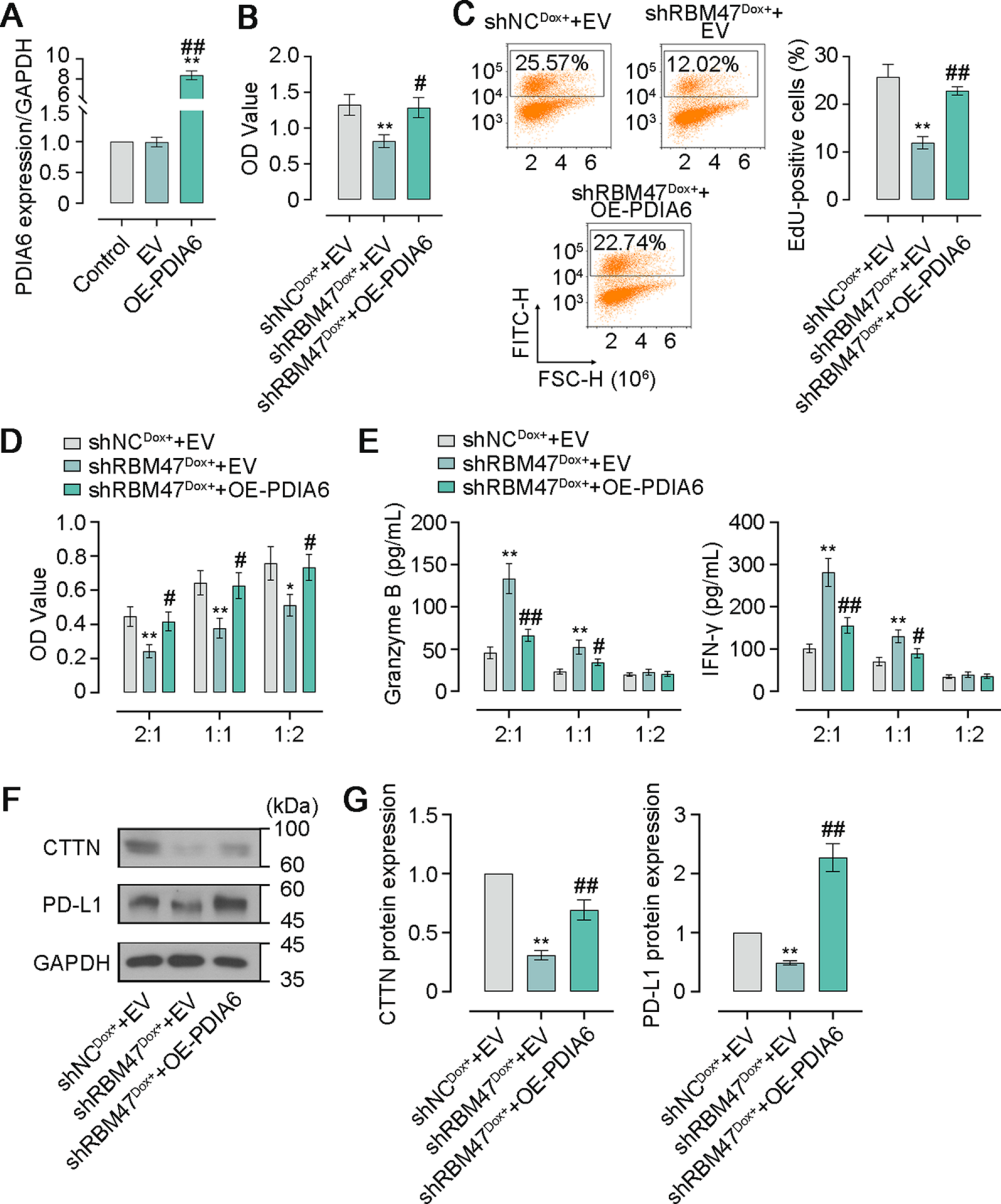
PDIA6 overexpression compromised the tumor-inhibiting effect of RBM47 knockdown on PC cells. **A** The mRNA level of PDIA6 expression in PC cells (normalized by GAPDH). **B** The CCK-8 assay for cell viability of PC cells. **C** Flow cytometry analysis for the percentage of EdU-positive PC cells. **D** The CCK-8 assay for cell viability of PC cells. **E** The content of Granzyme B and IFN-γ in supernatant of co-culture system consisting of NK and PC cells. **F** Western blot for PDIA6 expression. **G** Quantification of the protein levels of CTTN and PD-L1 expression in F. *p < 0.05, **p < 0.01 versus Control or shNC^Dox+^ + EV, ^#^p < 0.05, ^##^p < 0.01 versus EV or shRBM47^Dox+^ + EV

To investigate whether RBM47 impacted the development of PC via an effect on PDIA6, we first constructed the PDIA6 overexpression plasmid and then transfected RBM47-knockdown PC cells followed by Dox treatment for generating cells co-overexpressing RBM47 and PDIA6. The high transfection efficiency of the PDIA6 overexpression plasmid in PC cells was demonstrated by the dramatically increased PAIA6 mRNA level (Fig. 8A). CCK-8 assay showed that PDIA6 overexpression restored the decrease of cell viability caused by RBM47 knockdown (Fig. 8B). Similarly, the increased percentage of EdU-positive cells revealed the enhanced EdU incorporation in RBM47-knockdown PC cells after PDIA6 overexpression (Fig. 8C). After that, we conducted co-culture of NK 92 cells and PC cells co-overexpressing RBM47 and PDIA6 to further verify the impact on immune escape. The CCK-8 assay proved that PDIA6 overexpression eliminated the promoting effect of RBM47 knockdown on NK cell cytotoxicity (Fig. 8D). In addition, the increased concentrations of Granzyme B and IFN-γ in the co-culture supernatant induced by RBM47 overexpression were reduced by PDIA6 overexpression (Fig. 8E). In addition, RBM47 knockdown lowered the protein levels of CTTN and PD-L1 in PC cells, which was restored by PDIA6 overexpression to a certain extent (Fig. 8F, G).

### RBM47 knockdown alters cellular metabolites of PC cells

**Fig. 9:**
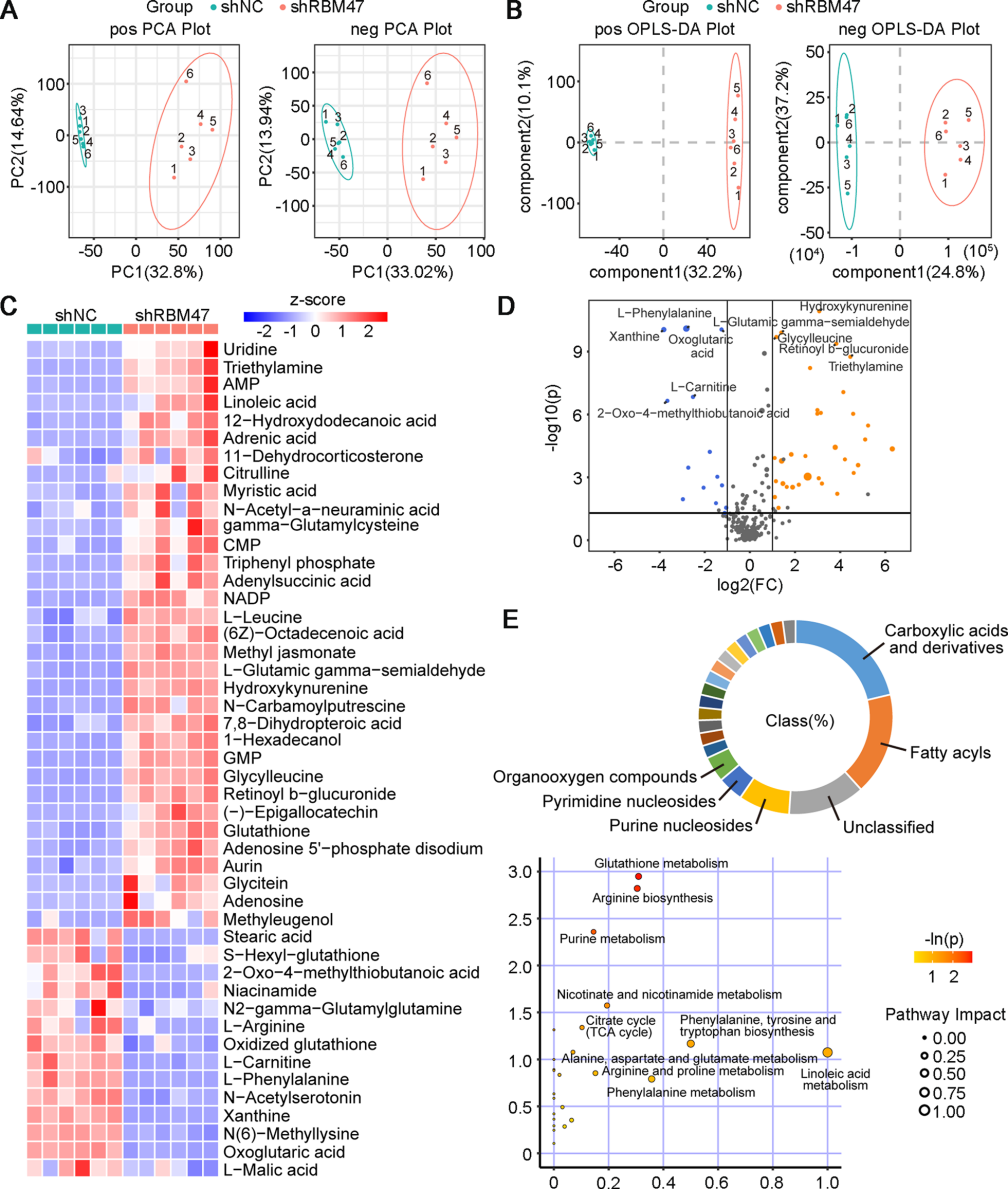
RBM47 knockdown changed the metabolite profile of PC cells. **A** The Principal Component Analysis (PCA) plot analyzed from non-targeted metabolomic analysis for metabolites extracted for PC cells. **B** Orthogonal projections to latent structures discriminant analysis (OPLS-DA) analyzed from non-targeted metabolomic analysis for metabolites extracted for PC cells. **C** The heat plot of differential metabolites from PC cells. **D** The volcano plot of differential metabolites from PC cells. **E** The percentage of metabolites in different class, and enriched KEGG pathways of differential metabolites from PC cells

Changes in cellular metabolic processes are considered as important hallmarks during the development of cancers [31]. Cell metabolism is a significant factor of cell proliferation of cancer cells [32]. A previous reported that glucose modulation caused by PKD1 contributed to PC cell proliferation [33]. Moreover, metabolism is able to regulate the tumor microenvironment, affecting the function of immune cells and further promoting immune evasion of tumor cells [34, 35]. Fructose 1,6-bisphosphatase 1 produced by tumor cells participated in the gluconeogenesis of NK cells and could decreased the NK cytotoxicity [36]. In view of the important role of cell metabolism in cancer progression, we performed the non-target metabolomics analysis to figure out metabolic changes in response to RBM47 knockdown. Principal component analysis (PCA) plots were generated from metabolites of PC cells detected in both positive and negative ion modes. The results showed that a clear separation of principal components between metabolites of RBM47-knockdown PC cells and their negative controls (Fig. 9A). Orthogonal projections to latent structures discriminant analysis (OPLS-DA) also indicated the distinct change in metabolites between the shNC group and the shRBM47 group (Fig. 9B). A total of 47 differential metabolites (33 upregulated and 14 downregulated) were identified and visualized in the heat map (Fig. 9C). The metabolite profile was presented in the volcano plot (Fig. 9D). Among the classifications of differential metabolites, carboxylic acids and derivatives and fatty acyls were the two major categories. These metabolites were further mapped to the KEGG pathway. Of note, several enriched metabolic pathways such as glutathione metabolism, arginine metabolism, and TCA cycle have been considered to be involved in cancer development (Fig. 9E).

## Discussion

RBM proteins contain two RNA recognition motif domains [25]. They are able to function as other RNA-binding proteins such as target RNA splicing, modification, and stability [37]. Several members in the RBM family participate in the development of cancer progression. RBM5 and RBM10 were determined as a tumor suppressor in lung cancer progression [38, 39]. RBM34 and RBM15 played an oncogenic function in hepatocellular carcinoma and PC progression [40, 41], respectively. In the current study, we found the higher RBM47 expression in PC tumor tissues by dataset analysis and explored the effects of RBM47 on PC progression. By subsequent in vitro and in vivo experiments, we found that RBM47 promotes malignancies of PC cells, including cell proliferation and xenografted tumor growth. Our study suggests that RBM47 might be a novel therapeutic target for PC treatment. Consistent with our findings, Xu et al*.* verified the inhibitory effect of RBM47 knockdown on proliferation of nasopharyngeal carcinoma cells [10]. A previous research also evidenced that RBM47 knockout mice exhibited less tumor formation after undergoing AOM/DSS treatment [9]. However, RBM47 was found to be downregulated in thyroid carcinoma and its overexpression inhibited proliferation of papillary thyroid carcinoma cells [42]. In addition, the inhibitory effects of RBM47 was previously reported in the development of non-small cell lung cancer [43], breast cancer [44], and hepatocellular carcinoma [45]. All above-mentioned results demonstrated that the dual role of RBM47 in different cancers. In the present study, we first identified RBM47 as a contributor of the development of PC.

Further, NK cells were co-cultured with PC cells to explore the immune escape of PC cells from NK cells. Our previous work and a previously published study selected the E:T ratios of 2:1, 1:1, and 1:2 to determine the killing effect of NK cells on PC cells with gene overexpression or knockdown [19, 46]. We referred to the previous research and used the E:T ratios of 2:1, 1:1, and 1:2 to explore whether RBM47 could affect the NK cells-mediated cytotoxicity. We found that, when NK cells/PC cells with a ratio 2:1, the higher RBM47 expression in PC cells suppresses the secretion of cytotoxic cytokines from NK cells, leading to the inhibition of NK-cell cytotoxicity. However, the similar effect of RBM47 overexpression was not observed in the co-culture system with a ratio 1:1 or 1:2. NK cells could mediate tumor cell killing in an E:T ratio-dependent manner [47]. An et al. evidenced that NK cell-mediated lysis of human chronic myelogenous leukemia K562 cells increased with increasing E:T ratio [48]. In the study of Cong et al., the E:T ratios of 2:1, 1:1, and 1:2 were used to explore the role of UQCRC1 in immune evasion of PC cells from NK cytotoxicity, and they found that the UQCRC1 overexpression promoted the cytotoxicity of NK cells against PC cells only the E:T ratios of 2:1, but not the ratios of 1:1, and 1:2 [46]. Our results were similar to the findings of the previous study. Moreover, actin cytoskeleton was associated with the immune evasion of tumor cells from cytotoxic T lymphocytes [49, 50]. Of note, F-actin is able to rapidly and massively accumulate near the IS and thereby lead to the immune escape of breast cancer cells from NK-mediated cytotoxicity, which termed as actin response (AR), causing the decreased intracellular level of Granzyme B [51]. Consistently, our findings revealed the aggregation of F-actin in PC cell after RBM47 overexpression, indicating that RBM47 might result in the AR and compromise the NK-cell attack. Importantly, CTTN could promote the stability and polymerization of F-actin filaments, thereby affecting the remodeling of actin cytoskeleton. We also found the increased CTTN expression and the interaction between CTTN and F-actin in PC cells. What’s more, CTTN overexpression reversed the suppressing-effect of RBM47 knockdown on F-actin accumulation, indicating that RBM47 promoted F-actin accumulation by affecting CTTN expression. Based on previous studies and our findings, we supposed that RBM47 led to AR in PC cells by upregulating CTTN expression and further remodeling F-actin cytoskeleton. Increased binding between PD-1 and PD-L1 in PC cells after RBM47 overexpression also revealed that RBM47 participated in immune evasion of PC cells through the PD-1/PD-L1 pathway.

The role of PDI family member PDIA6 in PC progression has been investigated in our previous work, which demonstrated that PDIA6 promoted PC progression and immune evasion of PC cells [19]. As one of the RBP family, RBM47 has the function of regulating downstream gene expression by sustaining their mRNA stability. Qin et al*.* reported that RBM47 positively regulated SNHG5 and its knockdown shorten the half-life of SNHG5, exerting an inhibitory effect on cell proliferation of papillary thyroid carcinoma cells [42]. RBM47 was reported to stabilize IFNAR1 mRNA and retard its degradation by binding to its 3′-UTR region [25]. We predicted and determined that RBM47 could bind to the PDIA6 3′-UTR region and sustain the PDIA6 mRNA stability. The rescue experiment of RBM47 knockdown and PDIA6 overexpression has further elucidated that effects of RBM47 on PC progression and immune surveillance was mediated by PDIA6.

Cellular metabolism tightly links to cancer development by regulating proliferation and immune evasion of cancer cells. Metabolomics data revealed the distinct metabolite profile between shNC-transfected PC cells and shRBM47-transfected PC cells. Importantly, some upregulated metabolites, such as linoleic acid and methyl jasmonate, in RBM47-knockdown PC cells have been well-established to take part in the risk of cancer occurrence. Linoleic acid could inhibit the proliferation of human liver cancer SK-HEP-1 cells and human prostate cancer LNCaP cells [52]. Similarly, methyl jasmonate was found to suppress the cell proliferation in different types of cancer [53]. In addition, further KEGG enrichment analysis revealed that altered metabolites might be associated with immune cells. TCA cycle provides energy for the activation of immune cells and its-produced metabolites modulate immune system [54, 55]. Arginine metabolism was identified as the key regulator for the immune cell function [56]. Nevertheless, the exact roles of these metabolites in proliferation and immune evasion of PC cells still require more experiments to verify.

Collectively, our findings evidence that RBM47 affects PC development by regulating PDIA6-medaited PC cell proliferation and immune escape from cytotoxic NK cells and altering cellular metabolites, providing the novel therapeutic target for PC treatment. However, there are some limitations in the present study. Firstly, given the important role of RBM family proteins in RNA processing and the strong link between RNA translation and cancer progression, we paid our attention to the RBM family members. Following that, RBM47 was selected for further study according to the association between gene expression and tumor immunity. However, other DEGs also has the potential to affect the development of PC. Therefore, our study cannot rule out the possibility that other DEGs might play a role in PC progression. Secondly, we found that RBM47 could regulate CTTN-related F-actin accumulation and the binding between PD-1 and PD-L1, thereby affecting immune evasion of PC cells from NK cells. However, the mechanism underlying RBM47 upregulated the expression of CTTN and PD-L1 has not been previously reported. Thirdly, we analyzed the metabolic status of PC cells after RBM47 knockdown by metabolomics, to provide more evidence that RBM47 regulated the malignancy of PC cells. However, this study did not further verify the effects of these metabolites on the proliferation and immune escape of PC cells with RBM47 overexpressing or knockdown. Furthermore, in the current study, we found that RBM47 could bind to the PDIA6 mRNA and thereby regulate PC cell proliferation and immune evasion. However, it is unknown whether the effect of RBM47 on cancer cell metabolism is related to PDIA6. Therefore, more experiments need to be performed to investigate the in-depth mechanism by which RBM47 affects behaviors of PC cells in the future.

## Conclusion

RBM47 was upregulated under the pathological state of PC. RBM47 promoted PC progression, manifested as cell proliferation and immune evasion from NK cells. The mechanism by which RBM47 affected immune evasion of PC cells might be caused by the aggregation of F-actin. RBM47 could bind to the 3′-UTR region of the PDIA6 mRNA and upregulated the PDIA6 expression. PDIA6 mediated the tumor-promoting effects of RBM47 on PC cells. Metabolomics analysis revealed the significantly altered metabolites in RBM47 knockdown PC cells compared with negative control cells. Taken together, RBM47 might upregulated the PDIA6 expression and changed cellular metabolism in PC cells, thereby promoting the development of PC.

## Supplementary Information

**Additional file 1: Table S1.** Relationship between the RBM47 expression and clinicopathological characteristics in patients with PC. **Fig. S1.** Immunohistochemistry staining for the RBM47 expression in tumor tissues of patients with PC. **Fig. S2.** The correlation between the RBM47 expression and the NK cell infiltrate in PC. **Fig. S3.** RBM47 knockdown inhibited the binding between PD-1 and PD-L1 in PC cells

## Data Availability

Data are available from the corresponding author upon reasonable request.

## Abbreviations

PC: Pancreatic cancer
RBM47: RNA-binding motif protein 47
RBP: RNA-binding protein
NK: Natural killer
IS: Immune synapse
GSCA: Gene Set Cancer Analysis
PDIA6: Protein Disulfide Isomerase Family A Member 6
PDI: Protein disulfide isomerase
UTR: Untranslated region
GEPIA: Gene Expression Profiling Interactive Analysis
PAAD: Pancreatic adenocarcinoma
DEGs: Differentially expressed genes
FC: Fold change
KEGG: Enriched Kyoto Encyclopedia of Genes and Genomes
GO: Gene ontology
BP: Biological process
CC: Cell component
MF: Molecular function
IHC: Immunohistochemistry
BSA: Bovine serum albumin
DAB: 3,3′-Diaminobenzidine
FBS: Fetal bovine serum
RPMI: Roswell Park Memorial Institute
MEM: Minimum essential medium
Dox: Doxycycline
CCK: Cell counting kit
OD: Optical density
PBS: Phosphate buffer saline
qRT-PCR: Quantitative real time-PCR
LDH: Lactatedehydrogenase
IP: Immunoprecipitation
ELISA: Enzyme linked immunosorbent assay
RIP: RNA-binding protein immunoprecipitation
VIP: Variable importance in the projection
SD: Standard deviation
E:T: Effector:target
PCA: Principal component analysis
OPLS-DA: Orthogonal projections to latent structures discriminant analysis
AR: Actin response
SNHG5: Small nucleolar RNA host gene 5
