# Supplementary material for: RBM47 promotes cell proliferation and immune evasion by upregulating PDIA6: a novel mechanism of pancreatic cancer progression
Source: J Transl Med. 2024 Dec 31;22:1164. doi: 10.1186/s12967-024-05970-6 (PMC11687039; doi:10.1186/s12967-024-05970-6)
Supplement: Supplementary file 1 — Additional file 1: Table S1. Relationship between the RBM47 expression and clinicopathological characteristics in patients with PC. Fig. S1. Immunohistochemistry staining for the RBM47 expression in tumor tissues of patients with PC. Fig. S2. The correlation between the RBM47 expression and the NK cell infiltrate in PC. Fig. S3. RBM47 knockdown inhibited the binding between PD-1 and PD-L1 in PC cells [file 12967_2024_5970_MOESM1_ESM.docx]

**RBM47 promotes cell proliferation and immune evasion by upregulating PDIA6: A novel mechanism of pancreatic cancer progression**

Yihui Ma^1*^; Enjie Liu^1^; Huijie Fan^2^; Chenfei Li^1^; Pei Huang^1^; Meiying Cui^1^; Zhengyang Wang^1^; Jing Zhou^1^; Kuisheng Chen^1*^

^*^Corresponding author:

Yihui Ma: Department of Pathology, The First Affiliated Hospital of Zhengzhou University, No. 1, Jianshe East Road, Zhengzhou, China

E-mail: [mapathology@163.com](mailto:mapathology@163.com)

Kuisheng Chen: Department of Pathology, The First Affiliated Hospital of Zhengzhou University, No. 1, Jianshe East Road, Zhengzhou, China

E-mail: [chenksh2002@163.com](mailto:chenksh2002@163.com)

**Supplementary materials**

**Table S1** Relationship between the RBM47 expression and clinicopathological characteristics in patients with PC

| Characteristics | | RBM47 expression | | P-value |
| --- | --- | --- | --- | --- |
|  |  | High | Low |  |
| Age | ≤ 60 | 11 | 14 | 0.349 |
|  | > 60 | 17 | 13 |  |
| Gender | Male | 15 | 13 | 0.687 |
|  | Female | 13 | 14 |  |
| T stage | T1 | 2 | 7 | 0.016* |
|  | T2 | 5 | 10 |  |
|  | T3 | 21 | 10 |  |
| N stage | N0 | 18 | 18 | 0.853 |
|  | N1 | 10 | 9 |  |
| Tumor size | ≤ 3cm | 13 | 17 | 0.218 |
|  | > 3cm | 15 | 10 |  |
| Distant metastasis | Positive | 10 | 9 | 0.853 |
|  | Negative | 18 | 18 |  |
| Lymph node metastasis | Positive | 18 | 18 | 0.853 |
|  | Negative | 10 | 9 |  |

*: significant difference.

**
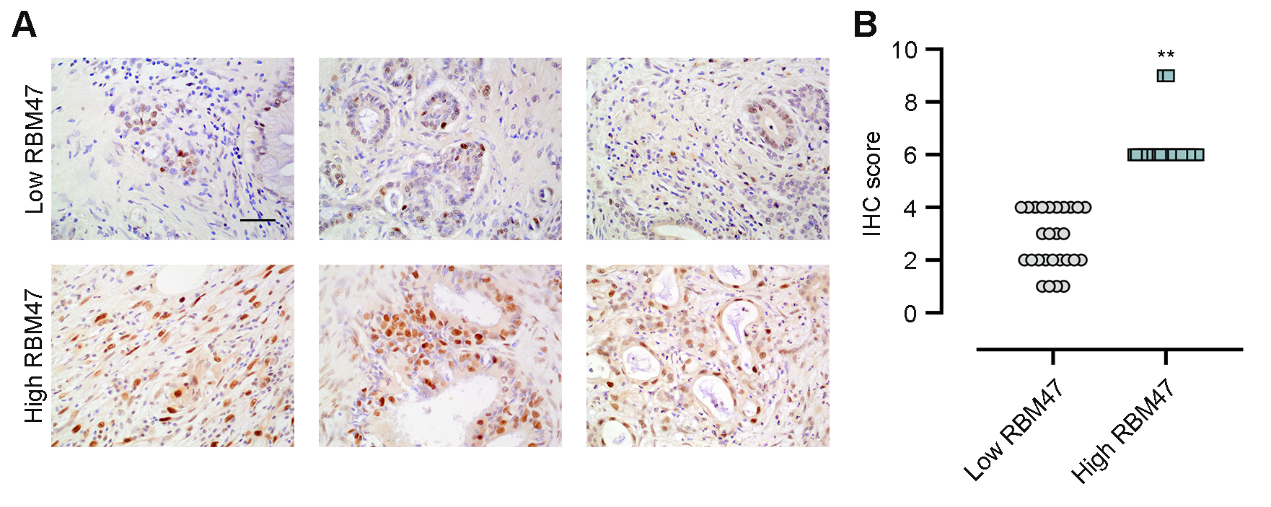
**

**Fig. S1** Immunohistochemistry staining for the RBM47 expression in tumor tissues of patients with PC.

(A) Representative image of tumor tissues with low RBM47 expression and high RBM47 expression. Scale bar = 50 μm. (B) IHC score of tumor tissues with low RBM47 expression and high RBM47 expression.

**
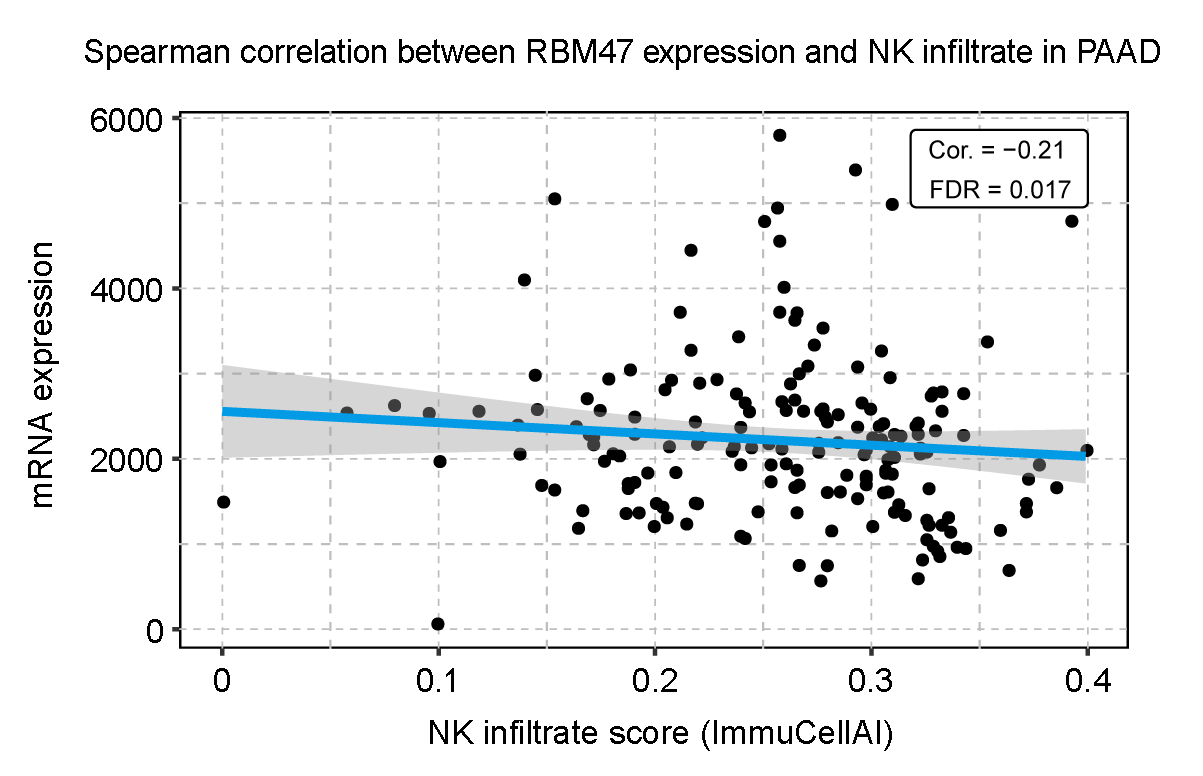
**

**Fig. S2** The correlation between the RBM47 expression and the NK cell infiltrate in PC

**
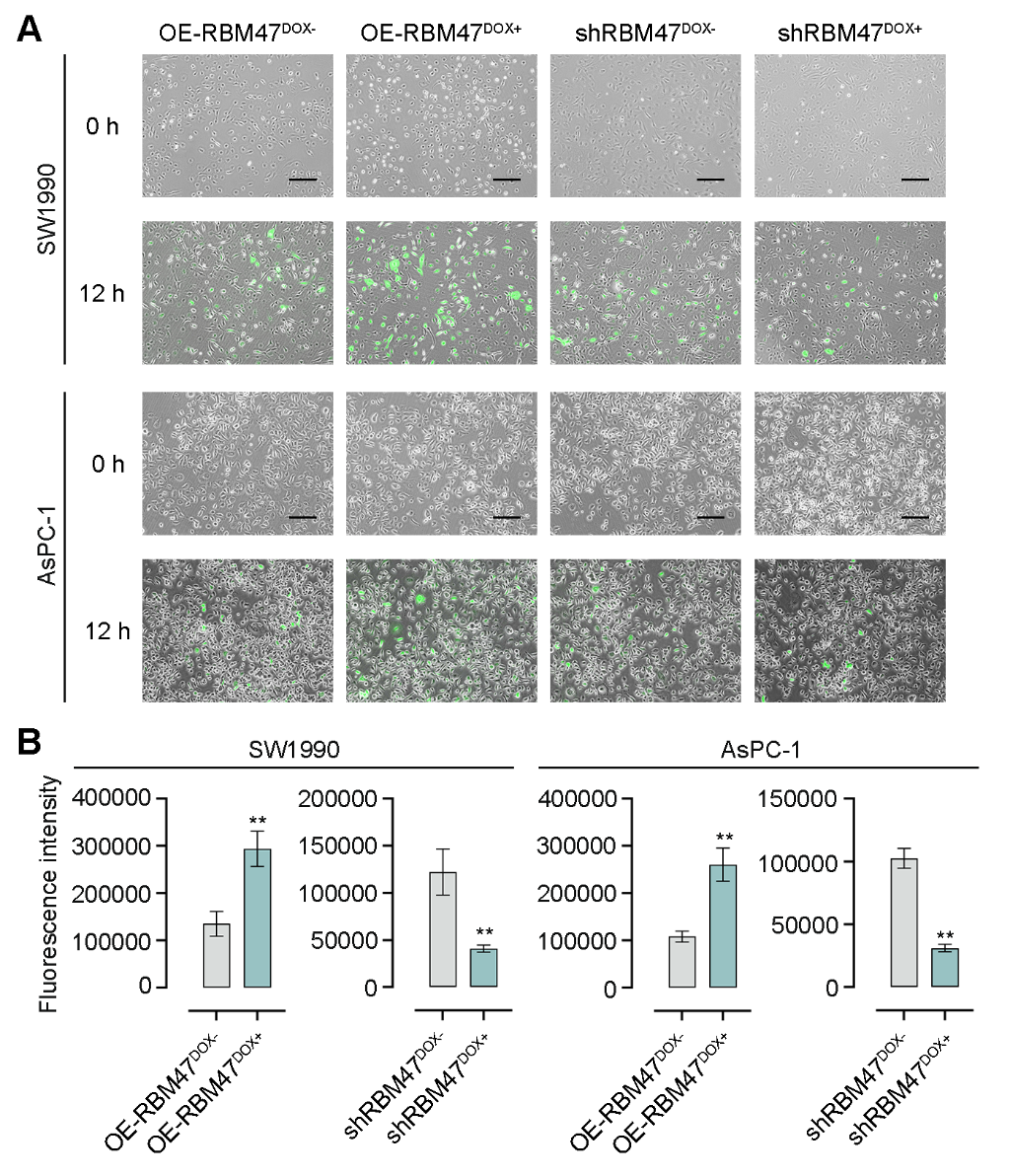
**

**Fig. S3** RBM47 knockdown inhibited the binding between PD-1 and PD-L1 in PC cells

(A) Images of the binding between FITC-labelled PD-1 and PD-L1 in PC cells. Scale bar = 200 μm. (B) Quantification of fluorescence intensity in Fig. S3A.
